# Quantitative Investigation of Hand Grasp Functionality: Thumb Grasping Behavior Adapting to Different Object Shapes, Sizes, and Relative Positions

**DOI:** 10.1155/2021/2640422

**Published:** 2021-11-15

**Authors:** Yuan Liu, Bo Zeng, Li Jiang, Hong Liu, Dong Ming

**Affiliations:** ^1^Tianjin University, Academy of Medical Engineering and Translational Medicine (AMT), Tianjin, China; ^2^Beijing Institute of Precision Mechatronics and Controls, Laboratory of Aerospace Servo Actuation and Transmission, Beijing, China; ^3^Harbin Institute of Technology, State Key Laboratory of Robotics and System, Harbin, China

## Abstract

This paper is the first in the two-part series quantitatively modelling human grasp functionality and understanding the way human grasp objects. The aim is to investigate the thumb movement behavior influenced by object shapes, sizes, and relative positions. Ten subjects were requested to grasp six objects (3 shapes × 2 sizes) in 27 different relative positions (3 *X* deviation × 3 *Y* deviation × 3 *Z* deviation). Thumb postures were investigated to each specific joint. The relative position (*X*, *Y*, and *Z* deviation) significantly affects thumb opposition rotation (Rot) and flexion (interphalangeal (IP) and metacarpo-phalangeal (MCP)), while the object property (object shape and size) significantly affects thumb abduction/adduction (ABD) motion. Based on the *F* value, the *Y* deviation has the primary effects on thumb motion. When the *Y* deviation changing from proximal to distal, thumb opposition rotation (Rot) and flexion (IP and MCP joint) angles were increased and decreased, respectively. For principal component analysis (PCA) results, thumb grasp behavior can be accurately reconstructed by first two principal components (PCs) which variance explanation ratio reached 93.8% and described by the inverse and homodromous coordination movement between thumb opposition and IP flexion. This paper provides a more comprehensive understanding of thumb grasp behavior. The postural synergies can reproduce the anthropomorphic motion, reduce the robot hardware, and control dimensionality. All of these provide a more accurate and general basis for the design and control of the bionic thumb and novel wearable assistant robot, thumb function assessment, and rehabilitation.

## 1. Introduction

The human hand is versatile in interactions with surrounding environments, showing a tremendous grasp functionality. Understanding the way humans grasp objects, completing the essential influence factor of human daily grasping, and knowing the specific kinematic adjusting associated with the influence factors are fundamental and important in neuroscience, robotics, prosthetics, medicine, and rehabilitation [1–3]. In our activities of daily lives (ADLs), people always grasp objects in different relative positions between hand and object, such as because of objects in different distances, task requirements, and space-constraints. It generally exists in ADLs (detailed explanation can be seen in the Electronic Supplementary Material (available here)). Human can successfully grasp various objects in different acceptable relative positions between human hand and object, termed as grasp tolerance [4] as a general grasp behavior which is necessary to be analyzed in detail in order to understand human grasp functionality comprehensively.

The aim of the series studies is to quantitatively investigate the human grasp functionality. However, human grasp functionality is a macrographic and vague description difficult to be understood and investigated in a detailed level. Therefore, we began our research from the review of qualitative study of hand grasp functionality in order to generalize the main definitions to embody the grasp functionality. After simultaneously considering these main definitions, we attempted to build one grasp paradigm for investigating hand grasp functionality quantitatively and comprehensively. Human grasp functionality has been extensively investigated. The first stream is based on the studies on grasp classification, as reported in Table 1. Schlesinger [5] first categorized human grasps into six types mainly based on the object and hand shape. The study is primarily related to the applications such as biomechanics, hand surgery, and rehabilitation [6]. The focus is on the grasp type. While to the same object, people may adopt different grasps according to action goals: hold object stably or impart the object motion. Napier et al. [7] categorized the grasps to power or precision grasp. In practice, the action goals can be reflected on the contact areas. Power grasp corresponds to wide contact area for stable holding, while the small contact area contributes to precision grasp. In this case, Kamakura et al. [8] later added the intermediate grasps for representing the postures with contact areas of finger-side aspect. In addition, based on the oppositional force direction applied between hand and object, Iberall et al. [9–11] divided the grasps into three categories: palm, pad, and side opposition. For a brief description, the virtual finger (VF) is proposed and defined as an abstract representation applying an oppositional force [11]. Then, Cutkosky [12] synthetically proposed a hierarchical tree of grasps, which begins with the two basic action goals suggested by Napier, moves down the tree, VF oppositional force and grasp type, totally lists 16 different grasps. For exploring the human grasping skills in more detail, Feix et al. [13] constructed a grasp taxonomy contained 33 human grasp types. The grasp type, action goal, and VF oppositional force are synthetically considered.

**Figure 1:**
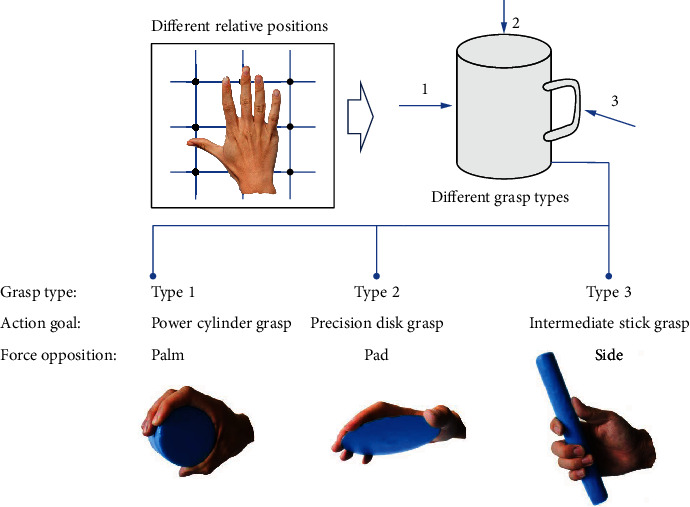
Different relative positions can lead to the diverse grasp types for the same object. Three main definitions of human grasp classification are represented and covered by adjusting relative positions of grasping.

In practice, in addition to the object shape and size, the relative position is another general factor that influences human grasping and leads to the grasp posture diversity. It can be seen from Figure 1 that different relative positions will result in different grasp positions leading to different grasp postures. The specific hand postures in different relative positions can be faithfully reconstructed, only when the relative positions are fully considered [14]. This indicates that the relative position is necessary to be particularly considered. Furthermore, as shown in Figure 1, induced by different relative positions, the grasp postures can represent and cover the three main definitions of grasp classification summarized in Table 1 including grasp type [5], action goal [7, 8], and oppositional force [9–11]. This indicates that the consideration of relative position can help people explore and understand human grasp functionality more comprehensively. More importantly, instead of the qualitative definitions summarized in Table 1, the relative position is a variable that can be actively quantified and precisely arranged in experiment, which can represent human grasp functionality more comprehensively. In addition, the relative position is a particular product of tolerance grasping that generally exists and plays an important role in ADLs (Electronic Supplementary Material). In our previous study to the analysis of hand and wrist postural synergies in tolerance grasping, the PCA analysis shows that the amount and dimension of information in tolerance grasping is increased by simultaneously considering the effects of relative position, object shape, and size. The first two PCs of tolerance grasping can only explain <65% (64.1%) of the information. This is obviously lower than other studies of hand kinematic synergies in related studies (~80% in grasp imagined objects [15], ~99% in reach-to-grasp for columnar objects [16], 70% in biometrics for secure identity verification [17], ~99% in precision grasping for cylinders of different size [18], ~70% in haptic exploration [19], ~80% in rapid grasping [20], ~88% in bimanual manipulation [21]). These results quantitatively demonstrate that, after considering the relative position effects on hand grasp, the tolerance grasping presented here can represent human grasp functionality more comprehensively.

For the study of human grasp functionality, the second stream is based on the kinematic studies of grasping. The relative positions between human hand and object are also seldom investigated as a general and particular influence factor on human grasp. Jeannerod proposed a sensorimotor control scheme to code the human grasping [22]. In visual guided grasping process, reaching to object is driven by the extrinsic object properties (e.g., object position), while grasping is determined by the intrinsic object properties (e.g., object size and shape). This concept leads to lots of researches; some of them look at the relationship between the hand kinematics and object properties including shape [15], size [23], fragility [24], texture [25], and mass [26, 27]. Some other researches focus on the behavior of the whole limb (shoulder and elbow), wrist and hand influenced by contextual task constraints; for example, the initial position of hands affected the hand reach trajectory [28]; end goal of the grasp action modified hand reach and grasp kinematics [29]. However, the effect of the relative positions on hand postures has seldom been investigated. Most of these studies focus on the human grasping in a specific context rather than the comprehensive representation of human grasp functionality. The grasp planning studies also support that relative position should be investigated as a particular influence factor on human grasp planning, such as reference frame adjusting from the eyes to the object [30] and the feedback control policy between the effector and the endpoint [31].

Altogether, the aforementioned investigation results strongly suggest that the relative positions should be investigated as a particular influence factor on hand grasping. At first, the relative positions as a general factor that influences human grasping in our ADLs are necessary to be considered and seldom investigated. Secondly, the acceptable relative position of human grasp can be quantitatively traversed within the tolerance range leading to the grasp posture diversity. The posture diversity can cover the main definitions of grasp classification. However, the object properties (e.g., shape, size, fragility, texture, and mass) are difficult to be traversed. Therefore, the protocol grasping the object in different relative positions can represent human grasp function more efficiently compared with grasping the objects of different properties. Thirdly, after the first simultaneous consideration of three general influence factors contained relative position, object shape, and size, the research can provide a more comprehensive understanding of hand grasp functionality.

In this paper, the relative position is given a particular attention as it is a general influence factor in human daily grasping and can more efficiently represent human grasp functionality. Moreover, in order to understand human grasp functionality more comprehensively, three general significant influence factors contained object shape, size, and relative position that are simultaneously considered as a whole for investigating the three factor effects on hand grasp. In addition, the particular opposition ability [32], neural [33, 34], musculoskeletal system difference [35], and independent motion [36] make thumb play a significant role in grasping. Therefore, we concentrate on the thumb grasp behavior in this paper. The detailed study specific to each joint can better clarify thumb movement behavior. The thumb kinematic behavior and synergies presented here are more representative and comprehensive to understand thumb general grasp functionality and the way human grasp objects. All of these provide a more accurate and general basis for the design and control of bionic thumb and novel wearable assistant robot, thumb function assessment, and rehabilitation.

## 2. Methods

### 2.1. Subjects

Ten healthy subjects of right-handed (24~27 years old, 8 men and 2 women) volunteered to take part in the experiment. Each subject is of good health and has no history of neurological or motor disorders. All participants were provided informed consent before the experiments, as required by the Declaration of Helsinki. The experiments were approved by the Ethical Committee of the university.

### 2.2. Experimental Setup and Protocol

**Figure 2:**
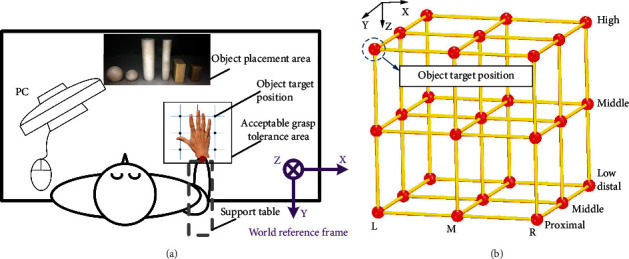
Experimental setup: (a) top view of the experimental setup; (b) 3D view of 27 relative positions (3 *X* deviation × 3 *Y* deviation × 3 *Z* deviation).

The subject sat in front of the table (Figure 2(a)). The elbow and wrist rested on the support tablet to make the forearm horizontal, the arm was oriented in the parasagittal plane passing through the shoulder, and the hand was in a pronated position. Right wrist through the wrist strap secured to the stationary bracket fixed to the experiment table, which was utilized to avoid the wrist transfer in Cartesian space, but the wrist can rotate in joint space to help hand reach to the object with different relative positions. In order to investigate hand grasp functionality more comprehensively and with a hand-centric consideration, the arm motion contribution to hand grasp is excluded by securing the wrist to the stationary bracket to avoid the wrist motion in Cartesian space. Wrist rotation is permitted in order to provide the perfect hand pose. The subject can accomplish the grasp successfully when the object is in different relative positions. The relative position in this paper is defined as the distance between the center of human wrist and object center of gravity. The object is placed in the target position shown in Figure 2(a). In order to facilitate understanding, Figure 2(b) is used to show the 3D view of the 27 target positions of the object. The relative position on the plane is achieved by placing the object at the target position as shown in Figure 2(a). The different relative heights (high, medium, low) between hand and objects were obtained by adjusting support tablet height. The support tablet and wrist strap were both fixed to the stationary bracket and could be adjusted to different height.

After the preexperiments, the acceptable grasp tolerance range was obtained and precisely arranged in 27 relative positions (3 *X* deviation × 3 *Y* deviation × 3 *Z* deviation). In preexperiments, the subject with the smallest hands was selected to be the first to explore the acceptable tolerance range. Once the range is determined, subsequent subjects try to grasp each object based on the determined range and are permitted to adjust the range according to their own hand ability. The range is only permitted to be narrowed in order to ensure that each participant can successfully accomplish the grasp within this range. There are three main requirements in experiment: (1) try to expand *X*, *Y*, and *Z* deviation of tolerance area as large as possible; (2) ensure that subject can successfully accomplish the grasp when the object is in each relative position of 27 relative positions; and (3) consider *X*, *Y*, and *Z* deviation equally rather than partial to one direction in the range exploration process. The top view and 3D view are shown in Figures 2(a) and 2(b), respectively. Within the grasp tolerance range, subjects can successfully grasp objects. The distance between the vertical lines and between the horizontal lines in the object target position area was 6 cm and 4.5 cm. The relative height between adjacent heights was 3 cm. The object is placed in 27 relative positions (3 *X* deviation × 3 *Y* deviation × 3 *Z* deviation) as shown in Figure 2(b). The shape, size, and weight of objects were selected (see Table 2) based on the Feix et al. [3], Zheng et al. [37], and Bullock et al. [6, 38] research results to high-effectively represent the objects we grasped in daily life.

Each subject was asked to grasp 6 different objects (3 shapes × 2 sizes) in 27 different relative positions (3 *X* deviation × 3 *Y* deviation × 3 *Z* deviation). Each object was grasped twice. In total, 324 trial (1 subjects × 6 objects × 27 relative positions × 2 repeats) across all six objects were performed by each subject over a period of ~2 h. Rest periods were interspersed among the trials to avoid fatigue. Subjects were instructed to firstly place the object from the object placement area to the target position with their left hand and then grasp the object with their right hand. After that, the subject was asked to hold the grasp posture three seconds for preliminary recording the posture. Then, subjects had to lift up the objects to ensure they could move objects successfully by the current grasp posture. Once completing the verification, the hand grasp posture was finally recorded. After accomplishing each grasp trial, the subject put the object back into the original position and began the next grasp trial until all trials were accomplished. No gesture constrains were given; the grasp postures were entirely decided by subjects under the premise of stable, nature, and comfortable grasping. No explicit constraints on movement velocity were given.

### 2.3. Recording of Human Hand Grasp

**Figure 3:**
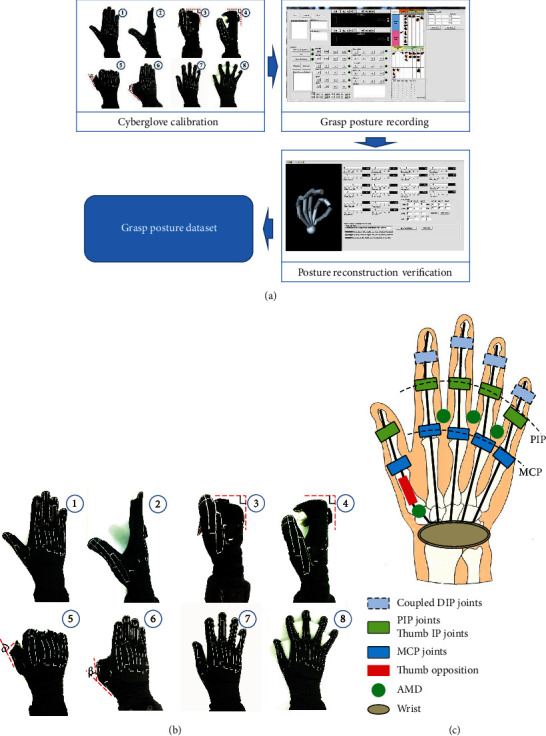
Calibration, recording, and reconstruction system: (a) calibration, recording, and reconstruction system; (b) Cyberglove calibration; (c) schema of the Cyberglove kinematic model contained 15 active joints.

In order to measure and record grasp posture accurately and efficiently, a recording and reconstruction system for human hand grasp was constructed (Figure 3(a)). Cyberglove III (Virtual Technologies, Palo Alto, CA) is used to measure the hand grasp posture, and PC is used to calibrate, record, and reconstruct the posture by the self-developed recording and reconstruction software (Figure 3(a)). At the beginning of experiment, the subject was asked to put on the Cyberglove. Then, a calibration on Cyberglove was carried on step by step (Figure 3(b) and Table 3) by a self-developed C++ software of calibration (Figure 3(a)). To guarantee the calibration accuracy, a key-posture Cyberglove calibration procedure (Figure 3(b)) was developed [39]. After finishing the Cyberglove calibration, the subject was asked to perform different kinds of grasp tasks. Simultaneously, hand postures were recorded by the C++ software. At last, to guarantee the accuracy of grasp posture, the hand grasp postures were verified by the self-developed posture reconstruction software (Figure 3(a)).

Hand grasp posture contained 15 joint angles that were actually recorded by Cyberglove III at a resolution of <0.1° and sampled at 100 Hz each. The following joint angles were measured (see Figure 3(b)): proximal interphalangeal (PIP) joints and metacarpo-phalangeal (MCP) joints of digits II-V, as well as the interphalangeal (IP) and MCP joints of the thumb (digit I), and the opposition rotation (Rot) of thumb, abduction/adduction (ABD) between each two adjacent fingers. As we concentrated the thumb posture in this paper, thumb Rot, IP, MCP, and ABD joint angles were used in statistical analysis. The step (2) figure of Figure 3(b) shows the opposition rotation of thumb Rot joint. The step (5) and (6) figures of Figure 3(b) show the flexion of thumb IP and MCP joints, respectively. The step (8) figure of Figure 3(b) shows the abduction of ABD joints.

### 2.4. Statistical Analysis

The mean of the two repeated trials was used in all statistical analyses. Five-factor ANOVA was performed to test the effect of independent factors on thumb posture. Independent factors were object shape (1-3), object size (1-2), *X* deviation (1-3), *Y* deviation (1-3), and *Z* deviation (1-3) between human hand and object. The dependent variables were four DoFs of thumb joint angle: opposition Rot of thumb, flexion/extension of IP and MCP joints of the thumb, and ABD of thumb CMC joint. Thumb grasp posture is investigated from three parts including opposition, flexion, and ABD movement. Principal component analysis (PCA) is applied to thumb posture data set, which is consisted of a 1620 × 4 matrix (10 subjects × 6 objects × 27 relative distances and 4 thumb kinematic DoF shown in Figure 3(c)). The PCA is used to obtain the synergy movement mode between four thumb joints and decrease the thumb movement degrees of freedom. Then, the clustering capacities of the retained PCs were explored. Finally, a five-factor ANOVA (object shape, object size, *X* deviation, *Y* deviation, and *Z* deviation) was performed for investigating the effects of the independent factors and interactions on each PC. The pairwise analysis between each two level group data under each significant influence independent factor is implemented to the joint angles of each joint among thumb four joints using Friedman's rank test after the Gaussian distribution test.

## 3. Results

### 3.1. Posture Analysis of Each Thumb Joint

#### 3.1.1. Thumb Opposition

**Figure 4:**
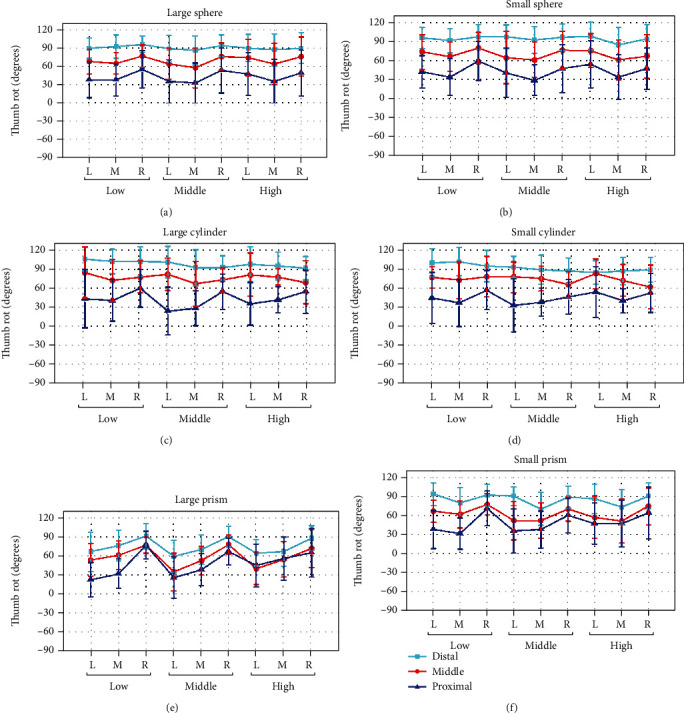
Thumb rotation joint angle influenced by the object shape, object size, *X* deviation, *Y* deviation, and *Z* deviation. L, M, and R represent the *X* deviation from left to right. Low, middle, and high represent the *Z* deviation from low to high. Distal, middle, and proximal represent the *Y* deviation from distal to proximal.

Figures 4(a)–4(f) show the thumb rotation joint angles averaged across subjects, when grasping the sphere, cylinder, and prism of large and small sizes in different relative positions. For grasping each object, thumb opposition all clearly varied as a function of *Y* deviation (proximal/middle/distal). The opposition angle increases as the *Y* deviation changing from proximal to distal. For grasping sphere (large/small) and cylinder (large/small) in the distal position of *Y* deviation, thumb opposition performs an approximately uniform mean angle about 90°.

While to *X* (L/M/R) and *Z* deviation (low/middle/high), the effect on thumb opposition is more obvious when object in the proximal position of *Y* deviation, especially for grasping prism (large/small). For grasping the object in the right position of *X* deviation (R), the thumb always performs a larger opposition angle than in left and middle position. For object shape, thumb opposition of prism grasping varied significantly compared with the other objects. However, the effect of the object size cannot be seen clearly from Figure 4.

#### 3.1.2. Thumb Flexion

Thumb MCP and IP joint flexion angle are used to represent the thumb flexion movement.

**Figure 5:**
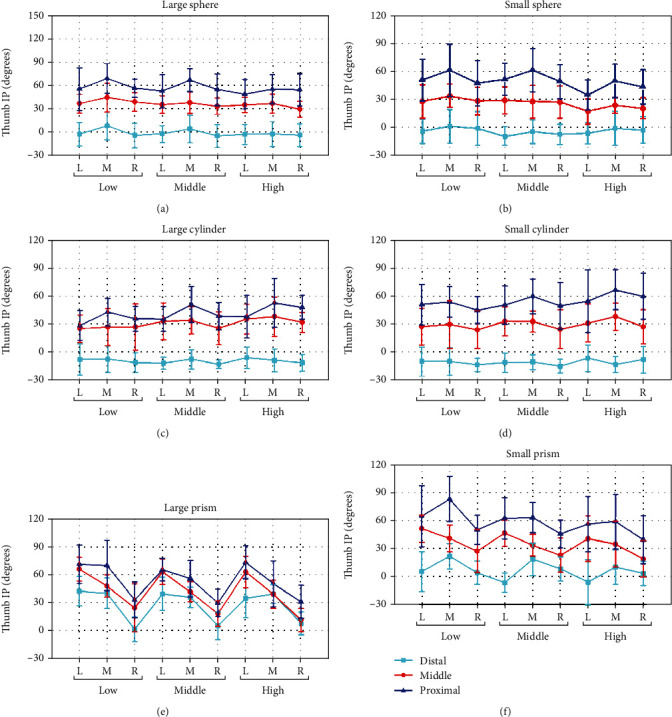
Thumb IP joint angle influenced by the object shape, object size, *X* deviation, *Y* deviation, and *Z* deviation. L, M, and R represent the *X* deviation from left to right. Low, middle, and high represent the *Z* deviation from low to high. Distal, middle, and proximal represent the *Y* deviation from distal to proximal.

*(1) Thumb IP Joint*. Figures 5(a)–5(f) show the thumb IP joint flexion angles averaged over subjects, when grasping the sphere, cylinder, and prism of large and small sizes in different relative positions. Similar to thumb opposition, for grasping each object, thumb IP joint flexion clearly varied as a function of *Y* deviation (proximal/middle/distal). The IP flexion angle decreases as the *Y* deviation changing from proximal to distal. For grasping sphere (large/small) and cylinder (large/small) in the distal position of *Y* deviation, thumb IP flexion performs an approximately uniform mean angle about 0°. For independent factor *X* (L/M/R) and *Z* deviation (low/middle/high), also similar to thumb opposition, the effect is more obvious when object is in proximal position of *Y* deviation, especially for grasping prism (large/small). For grasping the object in the right position of *X* deviation, thumb IP joint always performs a smaller flexion angle than in left and middle position in general. In terms of the effect of object shape, thumb IP flexion of prism grasping also varied significantly compared with the other objects. For object size influence, the distance between adjacent lines is larger for grasping the smaller objects, as shown in Figure 5. The effect of *Y* deviation is easier to be observed.

**Figure 6:**
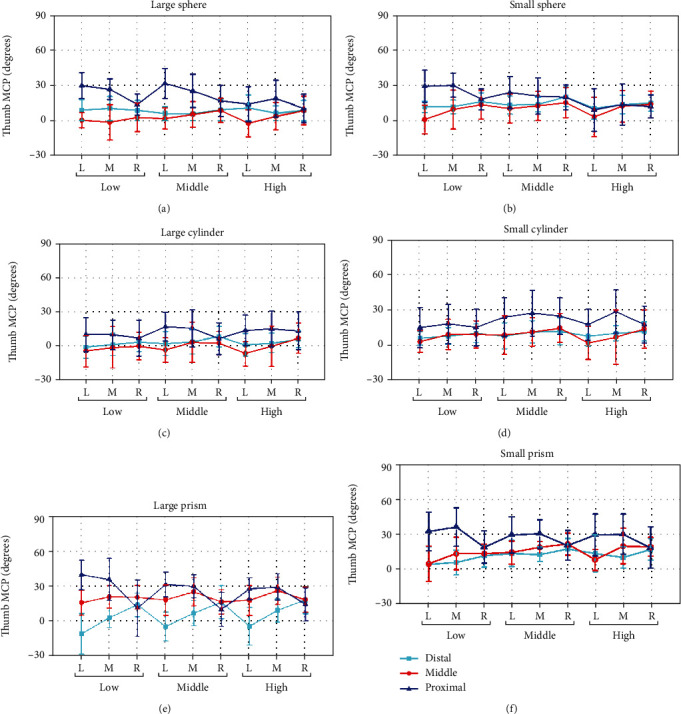
Thumb MCP joint rotation angle influenced by the object shape, object size, *X* deviation, *Y* deviation, and *Z* deviation. L, M, and R represent the *X* deviation from left to right. Low, middle, and high represent the *Z* deviation from low to high. Distal, middle, and proximal represent the *Y* deviation from distal to proximal.

*(2) Thumb MCP Joint*. Figures 6(a)–6(f) show the thumb MCP flexion joint angles averaged over subjects, when grasping the sphere, cylinder, and prism of large and small sizes in different relative positions. Compared with thumb opposition and IP flexion, the effect of relative position on MCP flexion is decreased, especially for *Y* deviation (proximal/middle/distal). For grasping the objects in proximal position of *Y* deviation, MCP joint has a larger flexion angle. Meanwhile, when the objects are in the right position, MCP flexion is small, while in the left position, the flexion is large. Compared with thumb opposition and IP flexion, the effect of object shape and size on MCP flexion is decreased as shown in Figure 6.

#### 3.1.3. Thumb ABD

**Figure 7:**
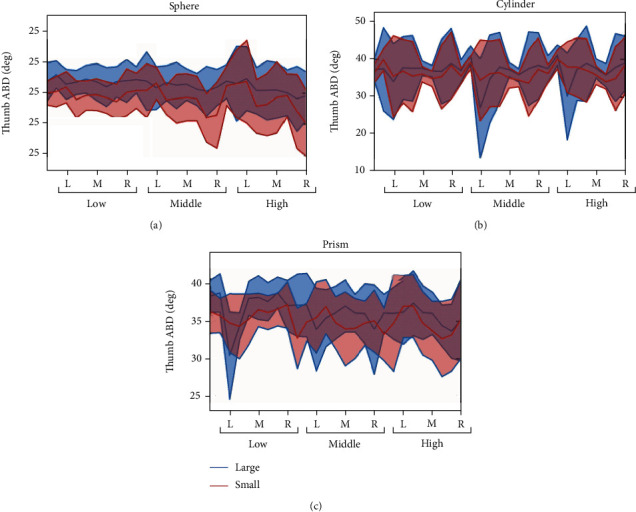
Thumb ABD joint flexion angle influenced by the object shape, object size, *X* deviation, *Y* deviation, and *Z* deviation.

For thumb ABD, the effect of each independent factor is smaller than thumb opposition and flexion. The reason is that the standard deviation of thumb ABD joint angle is only 5°, which indicates that the movement of thumb ABD joint is very limited in each grasp. However, we still find that ABD joint angle varies with different sizes. As shown in Figure 7, mean joint angle between large and small size objects varied significantly. Thumb ABD angle increases as the size is larger.

### 3.2. PCA on Thumb Posture

**Figure 8:**
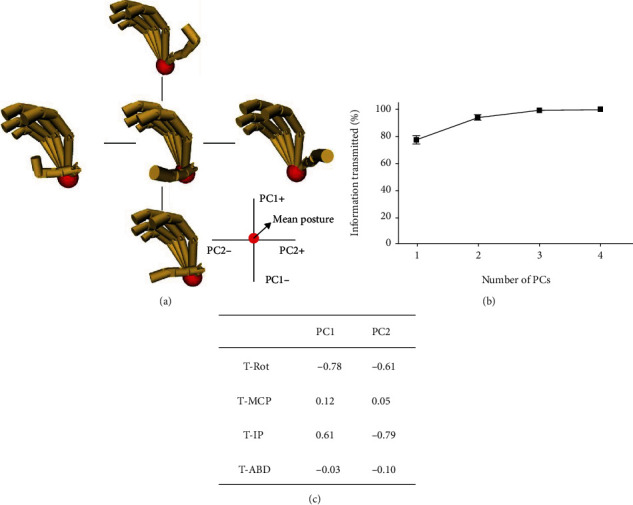
PCA on thumb posture: (a) thumb posture reconstruction by PC1-PC2; (b) information transmitted by PC1-PC4; (c) detailed element value of PC1-PC2.

**Figure 9:**
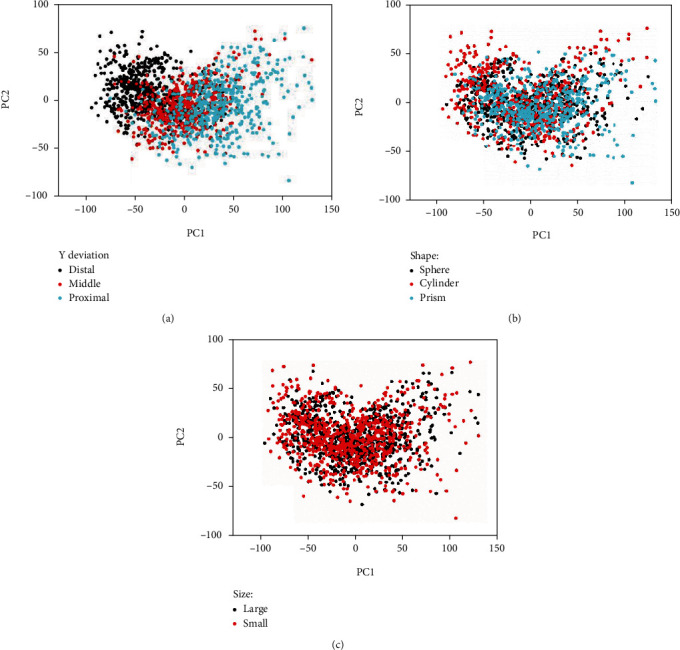
PCA of thumb posture. All 1620 samples across all subjects and experimental conditions are represented in the PC1-PC2 space. Each point represents one particular experimental condition for one subject (average of two repeated trials): (a) *Y* deviation (black distal in *Y* direction, red middle in *Y* direction, green proximal in *Y* deviation). Clearly, PC1 separates *Y* deviation; (b) shape (black sphere, red cylinder, green prism); (c) size (black large size, red small size).

The PCA of thumb posture across all ten subjects is shown in Figure 8.  Figure 8(a) shows the posture reconstruction by first two PCs. Meanwhile, as shown by the PC coefficients in Figure 8(c) (motion transmission ratio to each joint, positive and negative value represents the motion direction corresponding the Figure 8(a)), PC1 mainly represents the inverse movement of thumb opposition and IP flexion, and PC2 mainly represents synchronous movement of opposition and IP flexion. The PC1 accounted for 77.1% of the variance, while PC1-PC2 explained 93.8% of the variance. Thus, thumb posture can be accurately reconstructed by first two PCs according to the results of variance explanation rate.  Figure 9 shows the distribution of the 1620 samples in the space formed by PC1 and PC2. As shown in Figure 9(a), these samples can be categorized by *Y* deviation (proximal/middle/distal); it is apparent that PC1 differentiates between different *Y* deviations. However, the effect of object shape and size cannot be directly seen from Figures 9(b) and 9(c).

### 3.3. Five-Factor ANOVA and Pairwise Analysis

The five-factor ANOVA results are shown in Table 4 for quantifying the factor effects on thumb grasping. For each joint angle, it can be seen from Table 4 that the relative position (*X*, *Y*, and *Z* deviation) significantly affects thumb opposition (Rot joint) and flexion (IP and MCP joint), while the effect on thumb ABD motion is not significant. Based on the *F* value, the *Y* deviation has the primary effect on thumb motion. In addition, *X* and *Y* deviations have the most significant effect on IP joint, while *Z* deviation has the most significant effect on MCP joint. The object property (object shape and size) significantly affects all four thumb joints, contained thumb opposition (Rot joint), flexion (IP and MCP joint), and ABD. From the *F* value, the shape and size have the most significant effect on IP joint and MCP joint, respectively. Only the maximum value of *F* in all interaction factors is shown in Table 4. The interaction between shape and *X* deviation has the maximum value of *F* in all interaction factors for thumb opposition and IP joint flexion, while the interaction between *Y* and *X* deviation has the maximum effect on thumb MCP flexion and ABD motion.

For each PC of thumb posture, it can be seen from Table 4 that relative position (*X*, *Y*, and *Z* deviation) and object property (shape and size) significantly affect thumb PC1 motion (inverse movement of thumb opposition and IP flexion), while thumb PC2 motion (synchronous movement of opposition and IP flexion) is only significantly affected by *Y* deviation and shape. Only the maximum value of *F* in all interaction factors is shown in Table 4. For the interaction effects, the interaction between shape and *X* deviation has the maximum effect on PC1 motion, while PC2 motion is most obviously affected by shape and *Y* deviation interaction.

After pairwise analysis, we found that (1) the thumb opposition does not have a significant difference between sphere and cylinder object grasp (*P* = 0.49) and between low and high deviation (*P* = 0.8). (2) For the thumb flexion, thumb IP flexion is not significantly influenced by the object *Z* direction deviation between low and middle (*P* = 0.14) and the shape changing between cylinder and prism (*P* = 0.41). Thumb MCP flexion is not significantly influenced by the object *X* direction deviation between middle and right (*P* = 0.11) and the object *Z* direction deviation between low and high (*P* = 0.12). (3) For the thumb ABD, the influence of object changing from sphere to cylinder is not significant (*P* = 0.07). These results also can be verified by Figures 4–7. In addition, thumb motion is significantly influenced between any two levels among each independent factor with significant effect (*P* < 0.05).

## 4. Discussion

To the best of our knowledge, this paper first gives a particular attention to the relative position. On this basis, three general influence factors on human grasping contained object shape, size, and relative position are simultaneously considered as a whole for the first time to understand human grasp functionality more comprehensively. Moreover, due to the thumb unique and vital role, we separately analyzed thumb movement functionality in this paper. In this case, thumb functionality is investigated in detail and specific to each joint. The detailed study can better clarify the thumb grasp behavior and grasping. These results can provide a more accurate and comprehensive basis for thumb function assessment and rehabilitation. The extracted thumb postural synergies can help simply the robot thumb control, reduce the hardware needed actuators, and reproduce the anthropomorphic movement.

### 4.1. Thumb Grasping Behavior Adapting to Different Object Shapes, Sizes, and Relative Positions

The posture analysis results indicate that both thumb opposition (Figure 4) and flexion (Figure 5 for thumb IP joint, Figure 6 for thumb MCP joint) are significantly influenced by all factors contained *X*, *Y*, and *Z* deviation, object shape, and size, while ABD movement (Figure 7) is only significantly influenced by object property contained object size and shape. The main results were (1) for relative position effects, thumb opposition, and flexion varied significantly as a function of *X*, *Y*, and *Z* deviation (Figures 4–6, Table 4), but the thumb ABD is not sensitive (Table 4). Compared with *X* and *Z* deviation factor effects, *Y* deviation effects on the thumb opposition and flexion more significantly (Table 4). Specifically, as the increase of *Y* deviation, thumb opposition and IP flexion angle significantly increased (Figure 4) and decreased (Figure 5), respectively. When the object is in the proximal position of *Y* deviation, *X* and *Z* deviation affected thumb opposition and flexion more significantly. (2) For object shape and size effects, thumb opposition, flexion, and ABD are all varied significantly (Figures 4–7, Table 4). Especially for thumb ABD, only object shape and size impacted significantly (Table 4). (3) For PCA results (Figure 8), thumb posture can be accurately reconstructed by first two PCs which variance explanation ratio reached 93.8% (Figure 8(b)). PC1 mainly represents the inverse movement between opposition and IP flexion (Figure 8(a)), while PC2 mainly represents the thumb homodromous movement between opposition and IP flexion (Figure 8(a)). Both to PC1 and PC2, the movement ratio coefficients corresponding to ABD and MCP joint are small (Figure 8(c)); thus, the corresponding movement is limited, which is consistent with the research of hand natural movements [36].

**Figure 10:**
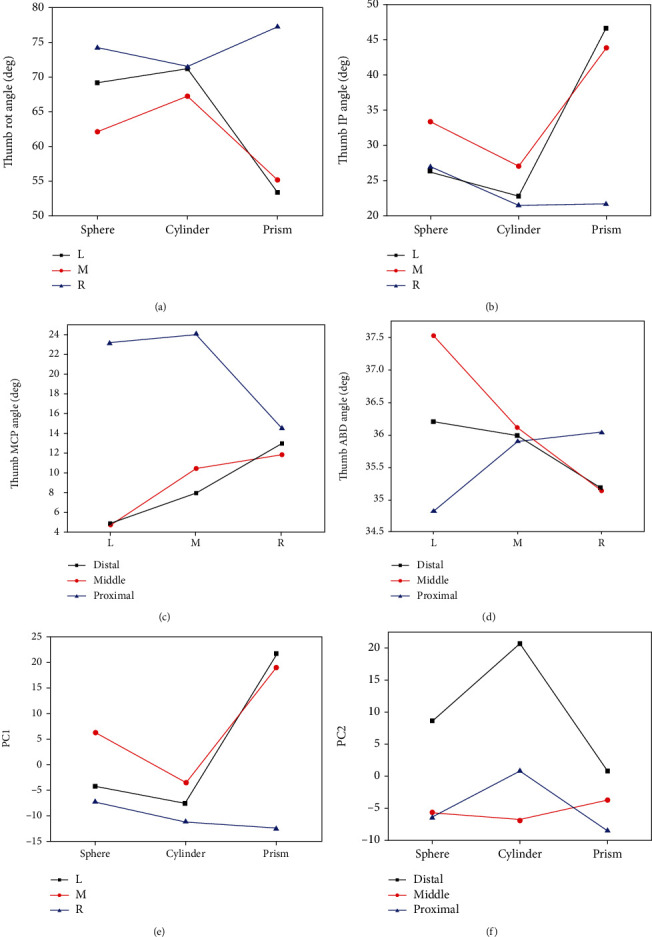
Two factor interaction plots: (a) shape versus *X* deviation for thumb Rot angle; (b) shape versus *X* deviation for thumb IP angle; (c) *X* deviation versus *Y* deviation for thumb MCP angle; (d) *X* deviation versus *Y* deviation for thumb ABD angle; (e) shape versus *X* deviation for PC1; (f) shape versus *Y* deviation for PC2.

Figures 10(a) and 10(b) illustrate the interaction effects between shape and *X* deviation on thumb Rot and IP joint angle. It can be seen from Figure 10(a) that the object shape effect on Rot joint angle performs an inverted triangle tendency when the object is placed at the right (R) position, which is different with other *X* deviations (performing a positive triangle tendency). Besides, the changing range in right deviation is also lower than in other *X* deviations. The influence of *X* deviation on each object shape grasp is as follows: prism > sphere > cylinder. In addition, as shown in Figure 10(b), the interaction effect tendency between shape and *X* deviation is similar. This is different from thumb Rot angle. For prism object, the effect of *X* deviation is obviously larger than other objects. Figures 10(c) and 10(d) illustrate the interaction effects between *X* and *Y* deviation on thumb MCP and ABD joint angle. It can be shown from the figure that the effect tendency of *X* deviation (L, M, and R) on thumb MCP and ABD joint angle is different when the object is at the proximal position of the *Y* deviation. When the object is at the distal and middle position of *Y* deviation, thumb MCP and ABD joint angle are increased and decreased when the object is placed from left to right, respectively. Figures 10(e) and 10(f) illustrate the interaction effects between shape and *X* deviation on PC1, and the interaction effects between shape and *Y* deviation on PC2. When the object is at the right (R) position of *X* deviation, the effect tendency of object shape on PC1 is different with other *X* deviation (L and M). In addition, when the object is at the middle (M) position of *Y* deviation, the effect tendency of object shape on PC2 is different with other *Y* deviations (distal and proximal).

### 4.2. Comprehensive Understanding of Human Grasp Functionality

After simultaneously considering the effects of relative position, object shape, and size, this paper is expected to provide a more comprehensive understanding of thumb grasp behavior. Firstly, the relative position as a general influence factor of daily grasp (see Electronic Supplementary Material) is firstly given a particular attention. Each object is grasped in 27 different relative positions. For each object, 27 different grasps are performed. In total, 3240 grasps (10 subjects × 6 objects × 27 relative positions × 2 repeats) are recorded. As shown in Figure 1, induced by different relative positions, the grasp postures can induce the grasp diversity to represent human grasp functionality more comprehensively. Secondly, three general influence factors (object shape, size, and relative position) are simultaneously considered as a whole for the first time. The amount and dimension of information in grasping is largely increased. The number of synergies required explaining grasp variance is obviously larger than other studies. Taking the first two synergies as the example, the variance explained rate is less than 65% [4] in this study, which is obviously lower than previous studies, such as grasping 57 imagined objects (about 80%) [15], 25 objects (about 70%) [17], 9 objects (about 88%) [21], and haptic exploration of 50 objects (about 70%) [19], and the comparison is also clearly reported in another grasp kinematic synergy study [40].

### 4.3. Towards the Wearable Assistant Robot Control and Design

After simultaneously considering three general influence factors on human grasping, the PCA results (Figure 8) in this paper are expected to represent thumb grasp behavior more comprehensively. The PCA results in this paper show that two motors (variance explanation ratio reached 93.8%, as shown in Figure 8(b)) are sufficient to reproduce thumb grasp behavior, although the thumb has more than five degrees of freedom. This is consistent with the thumb actuation configuration of current dexterous prosthetic hands (e.g., ilimb, Bebionic, and Vincent). The detailed element value of PC1-PC2 (Figure 8(c)) can help the dexterous prosthesis to reproduce the anthropomorphic thumb grasping motion, rather than the simple independent motion of two motors. In addition, the PCA results (Figure 8) can also serve the thumb mechanical hardware design for mechanically implementing thumb motion characteristics [41, 42]. The PCA of thumb posture (Figure 9) can help bionic thumb perform an anthropomorphic thumb motion driven by the relative position, object shape, and size, for accomplishing the precise object grasping task.

Furthermore, recently the supernumerary robotic limbs (SRL) have emerged in the field of robotics for compensating even enhancing the user's ability without replacing natural limbs [43]. The supernumerary robotic finger has been developed for hemiparetic upper limb rehabilitation [44–46]. Because the research in this area is just emerging, there are currently few theoretical reports on the design and control of SRL. Due to the thumb independence compared with other four fingers, the posture analyses to each thumb joint and postural synergies can provide a design basis to develop the novel wearable assistant robot independent with human body, such as supernumerary robotic finger. The detailed study specific to each joint can better help determine the actuation configuration [47, 48] in mechanism design and control [49]. The postural synergies can be used to reduce the hardware needed number of actuators and control dimensionality, reproduce the anthropomorphic movement to enhance the compatibility to the human workspaces [50], and provide a better body ownership sensory.

### 4.4. Limitations of This Study

Several limitations can be identified and should be provided. First, in order to provide a comprehensive investigation of hand grasp functionality, three main definitions (grasp type, action goal, and force opposition) are synthetically considered in our study. We built the tolerance grasping paradigm. The relative position, object shapes, and sizes were all simultaneously considered as a whole. The kinematic functionality is fully considered. However, the force consideration is seldom involved, e.g., the object weight impact on human grasping is not systematically investigated in this paper, and the finger joint stiffness and fingertip force distribution are also needed to further explored. Second, in order to efficiently investigate the object shape, size, and relative position impacts on human grasp, the objects were manufactured with the specific parameter requirements for high-effectively representing the objects we grasped in daily life. Therefore, they were not the real object we grasped in our daily lives. How to accurately match this paper results to a specific daily grasping task is also an interesting issue that we will pay attention to in the future.

## 5. Conclusion

In this study, three general influence factors on grasping contained relative position, object shape, and size are simultaneously considered in order to understand human grasp functionality more comprehensively. Thus, a general rather than particular understanding of thumb grasp behavior and the way human grasp objects is provided. Furthermore, the investigation of thumb behavior is performed to each specific joint in detail. The results indicate that thumb opposition and flexion varied significantly as a function of *X*, *Y*, and *Z* deviation, while the thumb ABD is only sensitive to object size and shape. Thumb grasp behavior can be accurately reconstructed by first two PCs which variance explanation ratio reached 93.8% and described by the coordination of inverse and homodromous movement between thumb opposition and IP flexion. All of these can contribute to a comprehensive understanding of thumb grasp behavior. The behavior analysis specific to each joint can better classify thumb movement characteristics and help design and control the bionic thumb and novel wearable assistant robot. The postural synergies can reduce the hardware and control dimensionality and reproduce the anthropomorphic movement to provide a better body ownership sensory.

## Figures and Tables

**Table 1:** Summary of selected precious studies on grasp classification.

Study	Definition	Description
Schlesinger [5]	Grasp type	Six typical postures are proposed to describe hand grasp functionality according to the object shape.

Napier [7] and Kamakura et al. [8]	Action goal	For the same object, the different action goals (power or precision) lead to different grasp postures. Power grasp serves to hold object stably, while precision grasp imparts the object motion [7]. The intermediate grasp was later added to represent the postures with contact areas of finger-side aspect [8].

Iberall et al. [9–11]	Oppositional force and virtual finger (VF)	For a given manual task, the grasp can be classified by the oppositional force exerted between virtual finger surfaces. Palm, pad and side opposition, mean oppositional force along a direction perpendicular, parallel and transverse to the palm, respectively.

Cutkosky [12]	Synthesis	Cutkosky [12] proposed a hierarchical tree of grasps, totally lists 16 different grasps. The grasp type, action goal, and VF oppositional force are synthetically considered.

Feix et al. [13]	Synthesis	Feix et al. [13] constructed a grasp taxonomy contained 33 human grasp types. The grasp type, action goal, and VF oppositional force are synthetically considered.

**Table 2:** Shape, size, and weight of the six grasping objects.

Shape		Size (mm)	Weight (g)
Sphere	Large	Diameter 80	300
	Small	Diameter 60	100
Cylinder	Large	Diameter 60; height 200	650
	Small	Diameter 40; height 200	300
Prism	Large	Length 80; width 40; height 100	300
	Small	Length 40; width 40; height 100	150

**Table 3:** The calibration joints and actual joint angles in each calibration step.

Step	Calibration joints	Actual joint angle
1	PIP and MCP joints of four digits and thumb ROT joint	0°
2	Thumb ROT joint	90°
	Thumb MCP joint	0°
3	MCP joints of four digits	90°
4	PIP joints of four digits	90°
5	Thumb IP joint	*α* = 10°, 30°, 50°, 70°
6	Thumb MCP joint	*β* = 10°, 30°, 45°, 60°
7	All ABD joints between adjacent fingers	15°
8	All ABD joints between adjacent fingers	30°

**Table 4:** *F* values of the ANOVA on the factor scores of each joint angle and PC of thumb posture.

Motion	Joint	Size	Shape	X deviation	Y deviation	Z deviation	Interaction
Opposition	Rot	*F*(1) = 6.95	*F*(2) = 13.36	*F*(2) = 32.67	*F*(2) = 363.62	*F*(2) = 5.52	*F*(4) = 10.4
Flexion	IP	*F*(1) = 14.77	***F*(2) =85.81**	***F*(2) =63.96**	***F*(2) =1189.44**	*F*(2) = 3.52	*F*(4) = 31.78
	MCP	***F*(1) =48.84**	*F*(2) = 52.69	*F*(2) = 9.79	*F*(2) = 165.55	***F*(2) =8.12**	*F*(4) = 29.9
ABD	ABD	*F*(1) = 21.23	*F*(2) = 12				*F*(4) = 5.92
PCA	PC1	*F*(1) = 16.42	*F*(2) = 57.15	*F*(2) = 66.34	*F*(2) = 1001.67	*F*(1) = 3.69	*F*(4) = 22.25
	PC2		*F*(2) = 19.97		*F*(2) = 78.23		*F*(4) = 11.24

*F*(*b*) = *F*(*a*, *b*), *a* = 1522. Only significant factors are shown in the table (*p*≦0.05). Only the maximum value of *F* in all interaction factors is shown in the interaction. Bold values indicated most obvious changing joint angle influenced by each factor.

## Data Availability

The datasets used and/or analyzed during the current study are available from the corresponding author on reasonable request.

## Supplementary Materials

Supplementary Materials Electronic Supplementary Material provides the supplementary explanation to the general existence in ADLs grasping of changing relative position.
