# Supplementary material for: Quantitative Investigation of Hand Grasp Functionality: Thumb Grasping Behavior Adapting to Different Object Shapes, Sizes, and Relative Positions
Source: Appl Bionics Biomech. 2021 Nov 15;2021:2640422. doi: 10.1155/2021/2640422 (PMC8608516; doi:10.1155/2021/2640422)
Supplement: Supplementary Materials — Electronic Supplementary Material provides the supplementary explanation to the general existence in ADLs grasping of changing relative position. [file 2640422.f1.pdf]

Applied Bionics and Biomechanics

Quantitative Investigation of Hand Grasp Functionality: Thumb Grasping Behavior Adapting to Different Object Shapes, Sizes and Relative Positions

(*Electronic supplementary material*)

Yuan Liu[1], Bo Zeng[2], Li Jiang[3], Hong Liu[3], Dong Ming[1]

[1]Academy of Medical Engineering and Translational Medicine
Tianjin University
Tianjin, 300072, China.

[2]Beijing Institute of Precision Mechatronics and Controls
Laboratory of Aerospace Servo Actuation and Transmission
Beijing, 100000, China.

[3]State Key Laboratory of Robotics and System,
Harbin Institute of Technology,
Harbin, Heilongjiang 150080, China.

This document includes:

- Supplementary explanation to the general existence in ADLs grasping of changing relative position

- Supplementary figure

-Supplementary references

Supplementary explanation to the general existence in ADLs grasping of changing relative position

In this section, we provide some typical scenes which indicating that human tolerance grasping generally exists in activities of daily lives (ADLs) as one kind of human essential grasp functionalities, which is necessary to be analyzed in a detailed level for understanding human grasp functionality more comprehensively.

Firstly, in daily life we often stand in a position to grasp the target objects that are placed at different distances. Two representative examples contained grasping the objects in market shelves and drawers are shown in Fig. S1 (a) and Fig. S1 (b), respectively. The different object target positions always make people grasp the object with different relative positions and perform different grasp postures [1]. In particular, when shopping in the supermarket or preparing food in the kitchen, we often grasp the object while scanning the products in shelves or preparing food, this kind of arbitrary reaching may lead to grasp the object in a larger position deviation. In these cases, human still can successfully grasp it. This indicates that the tolerance grasping generally exists in our daily lives.

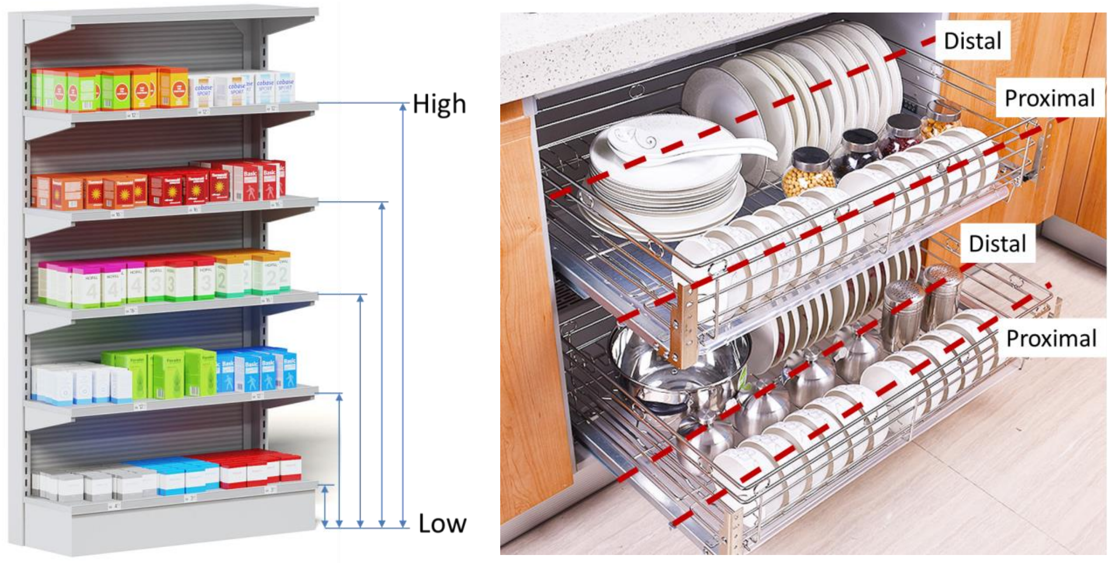

(a) (b)

Figure S1. Grasping the objects located in different positions. (a) Objects on the market shelves with different height, (b) bowls are located in the drawer with distal and proximal positions.

Secondly, for different task requirements, people often perform tolerance grasping. Fig. S2 shows that when the target objects which the wrenches act on is in different distances, people need to adjust different postures to grasp wrench in different task-requirement situations with different relative positions between hands and wrench center of gravity, performing different postures as shown in Fig. S2.

Thirdly, for space-constraint, people often have to perform the compromised postures to complete the task. People need to adjust the different compromised postures to grasp screwdriver in different space-constraint situations with different relative positions between hands and screwdriver center of gravity, performing the ability of grasping tolerance as shown in Fig. S3.

These two cases show the tolerance grasping generally exists in the tool using.

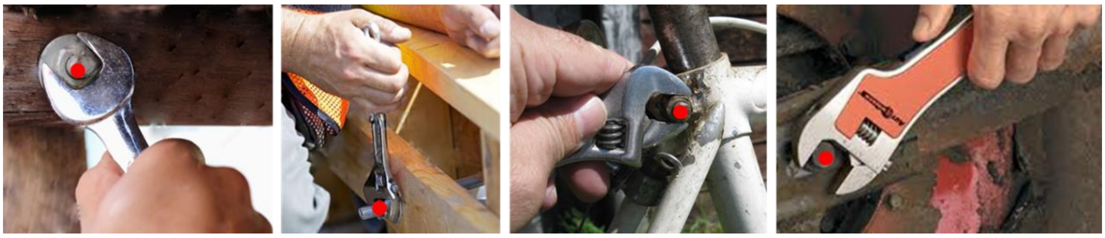

Figure S2. Task requirements for people to grasp the tool in different relative positions. Red point represents the repaired target objects. When the target objects which the wrenches act on is in different distances, people need to change different postures to grasp the wrench in different relative positions, showing the grasp tolerance ability for the tool using.

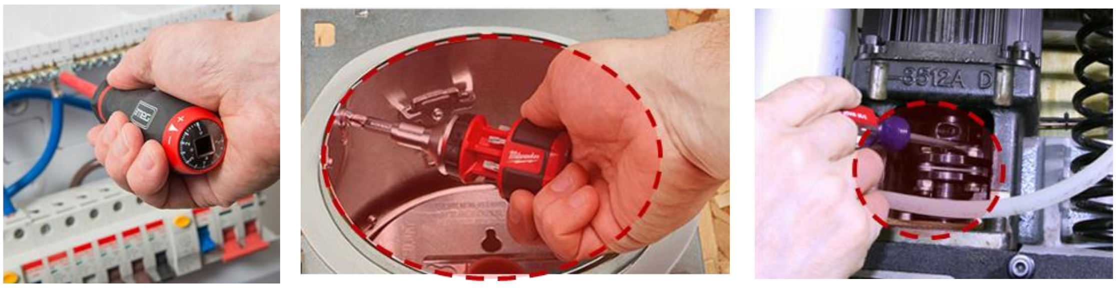

(a) (b) (c)

Figure S3. Space-constraint for people to grasp the same object in different relative positions. The red area represents the space-constraint situation. (a) Perfect posture without space-constraint, (b) compromised posture I with space-constraint, (c) compromised posture II with space-constraint.

Supplementary references

[1]. Touvet, François, et al. "Grasp: combined contribution of object properties and task constraints on hand and finger posture." Experimental brain research 232.10 (2014): 3055-3067.
